# Irreversible electroporation combined with immunotherapy *versus* irreversible electroporation alone for locally advanced pancreatic cancer: a systematic review and meta-analysis

**DOI:** 10.31744/einstein_journal/2026RW1656

**Published:** 2026-04-07

**Authors:** Miriana Mariussi, Laura Costa de Oliveira Lima, Mariano Gallo Ruelas, Victor Arthur Ohannesian, Bruno Murad, Guilherme Strieder de Oliveira, Luiza Giuliani Schmitt, Giovanni Brondani Torri, Stephan Altmayer, Priscila Mina Falsarella, Pedro Luiz Serrano Usón, Rodrigo Gobbo Garcia

**Affiliations:** 1 Hospital Israelita Albert Einstein São Paulo SP Brazil Hospital Israelita Albert Einstein, São Paulo, SP, Brazil.; 2 Pontifícia Universidade Católica de Campinas Campinas SP Brazil Pontifícia Universidade Católica de Campinas, Campinas, SP, Brazil.; 3 Instituto de Investigación Nutricional Lima Peru Instituto de Investigación Nutricional, Lima, Peru.; 4 Hospital Israelita Albert Einstein Faculdade Israelita de Ciências da Saúde Albert Einstein São Paulo SP Brazil Faculdade Israelita de Ciências da Saúde Albert Einstein, Hospital Israelita Albert Einstein, São Paulo, SP, Brazil.; 5 University of Wisconsin-Madison Madison WI United States University of Wisconsin-Madison, Madison, WI, United States.; 6 Hospital de Clínicas de Porto Alegre Porto Alegre RS Brazil Hospital de Clínicas de Porto Alegre, Porto Alegre, RS, Brazil.; 7 UT Southwestern Medical Center Dallas TX United States UT Southwestern Medical Center, Dallas, TX, United States.; 8 UF Health Shands Hospital Gainesville FL United States UF Health Shands Hospital, Gainesville, FL, United States.; 9 Stanford University School of Medicine Palo Alto CA United States Stanford University School of Medicine, Palo Alto, CA, United States.

**Keywords:** Electroporation, Pancreatic neoplasms, Immunotherapy, Ablation techniques

## Abstract

**Objective:**

The aim of this meta-analysis was to determine the efficacy and safety of percutaneous irreversible electroporation combined with immunotherapy compared with irreversible electroporation alone in patients with locally advanced pancreatic cancer.

**Methods:**

We systematically searched Embase, Cochrane Central Register of Controlled Trials, and PubMed/Medline for relevant studies. The outcomes of interest were progression-free survival, overall survival, carbohydrate antigen 19-9 (CA 19-9) levels, and adverse events. Progression-free survival and overall survival were assessed using pooled hazard ratios (HR), odds ratios (OR) were used for adverse events, and mean differences (MD) for CA 19-9.

**Results:**

Four studies involving 310 patients were included in the pooled analysis. Irreversible electroporation combined with immunotherapy significantly prolonged progression-free survival compared with irreversible electroporation alone (hazard ratio [HR], 0.56; 95%CI=0.39 – 0.80; p<0.01; I^2^=10%). Additionally, patients who received irreversible electroporation plus immunotherapy achieved a greater overall survival compared with irreversible electroporation alone (HR=0.52; 95%CI=0.37 – 0.73; p<0.01; I^2^=0%). The pooled results for CA 19-9 showed significantly lower levels in patients receiving irreversible electroporation and immunotherapy compared with those receiving irreversible electroporation alone (MD: −70.18U/L; 95%CI=-121.07 – −19.29; p<0.01; I^2^=98%). No significant difference in the occurrence of adverse events such as nausea and vomiting (OR=1.58; 95%CI=0.71 – 3.49; p=0.26; I^2^=0%) and gastroparesis (OR=0.88; 95%CI=0.23 – 3.40; p=0.85; I^2^=0%) was not observed between the groups.

**Conclusion:**

Combined therapy using percutaneous irreversible electroporation and systemic immunotherapy offers a safe and effective treatment approach for locally advanced pancreatic cancer, with irreversible electroporation potentially enhancing the efficacy of systemic immunotherapy in combined applications.

**Prospero database registration:**

ID CRD42024562216.

## INTRODUCTION

Pancreatic ductal adenocarcinoma (PDAC) is the most common malignant neoplasm of the pancreas and its incidence and mortality rates have increased in recent years.^(1)^ Pancreatic ductal adenocarcinoma is categorized into three non-metastatic categories based on resectability status: resectable, borderline resectable, and locally advanced pancreatic cancer (LAPC). Surgery remains the only potentially curative treatment, yet only 15% of patients present with resectable disease. In these patients, margin-negative resection followed by adjuvant chemotherapy is associated with 5-year survival rates of approximately 20%.^(2)^ Nearly 30% of cases are classified as LAPC at diagnosis, which involves a locoregional tumor extending to adjacent vasculature or structures without distant metastasis. These tumors are considered unresectable, with an overall survival of 9–13 months.^(3)^

Management of LAPC focuses on symptom control such as pain related to celiac plexus involvement, digestive obstruction, and nutrition-related weight loss. For patients with good or intermediate performance status (PS), first-line treatment typically consists of induction chemotherapy followed by chemoradiation or stereotactic body radiotherapy (SBRT). Targeted therapy based on molecular profiling may be considered as a subsequent therapy in patients with disease progression and poorer PS.^(4)^ Pancreatic cancer is largely refractory to immunotherapy and is considered an immune-cold tumor.^(5)^ In a Phase II randomized clinical trial, O’ Reilly et al. reported poor response rates with anti-PD-1 alone or anti-PD-1 combined with anti-CTLA-4 drugs in PDAC.^(6)^ In contrast, Chen et al. reported significantly improved responses with combined anti-PD-1, anti-CTLA-4, and SBRT in patients with PDAC.^(7)^ Despite the availability of systemic therapy options, tumor responses remain unsatisfactory, highlighting the potential role of minimally invasive ablation techniques for focal tumor destruction.^(8)^

Irreversible electroporation (IRE) is a non-thermal ablation technique that delivers high-voltage electrical pulses to induce cell death through apoptosis. Because it is nonthermal, IRE preserves surrounding tissue structures, including major blood vessels, bile ducts, and the intestines. IRE also triggers significant antigen release and T-cell activation post-therapy, providing potential synergistic effect when combined with immunotherapy to enhance targeting and destruction of tumor cells.^(9–11)^

## OBJECTIVE

Therefore, the aim of this systematic review and meta-analysis was to determine the efficacy and safety of percutaneous irreversible electroporation combined with immunotherapy compared with irreversible electroporation alone in patients with locally advanced pancreatic cancer.

## METHODS

### Search strategy

We systematically searched the MEDLINE, Embase, and Cochrane Library databases for articles published until July 2025. The search strategy included terms such as "irreversible electroporation," "immunotherapy," and "locally advanced pancreatic cancer." The MeSH and input terms were adapted for the selected databases, combining terms with Boolean connectors (OR and AND) and conforming to the syntax rules in each database. Duplicate articles were manually excluded. References from all included studies, previous systematic reviews, and meta-analyses were also manually searched for additional studies, and reference manager software, Zotero^®^ (Version 7.0.3), was used.^(12)^ The search strategy for each database is presented in Table 1S, Supplementary Material. Our study was conducted in accordance with the Preferred Reporting Items for Systematic Reviews and Meta-Analyses (PRISMA) guidelines and the Cochrane Handbook for Systematic Reviews of Interventions.^(13,14)^

### Study selection

Two reviewers independently assessed the initial search results to identify studies that met the eligibility criteria based on their titles and abstracts. The selected studies then underwent full-text assessment by the same reviewers, who made the final selection based on the inclusion and exclusion criteria. Any disagreements were resolved through discussion among all authors, with the lead reviewer making the final decision, if necessary.

### Eligibility criteria

Inclusion in this meta-analysis was restricted to studies that met the following eligibility criteria: (1) enrolled patients with LAPC, (2) compared IRE combined with systemic immunotherapy with IRE alone, (3) were clinical trials or observational studies, and (4) reported any of the outcomes of interest.

We excluded studies that: (1) used ablation methods other than IRE; (2) included intratumoral immunotherapy as treatment; (3) had no Control Group; (4) had overlapping patient populations, retaining only the study with the highest number of patients; (5) were reviews, case reports, editorials, correspondences, comments, or meeting abstracts; (6) used a murine animal model; (7) did not have available full texts; and (8) were written in languages other than English.

### Data extraction of study characteristics

Two reviewers independently extracted relevant data from the selected studies in a standardized form. The extracted data included study characteristics and demographic data such as the first author, year of publication, study location, total sample size, sample size of the Control Group (irreversible electroporation alone arm), sample size of the Intervention Group (irreversible electroporation plus immunotherapy arm), immunotherapy drug regimen, sex and age (median and/or average) of the sample population, study design, single- or multi-center setup, follow-up time, and tumor diameter (Table 1). Any disagreement between the two reviewers was resolved by consensus with the assistance of a third reviewer.

**Table 1:** Main characteristics of studies included in the review

First author	Year of publication	Location (Country)	Single/Multi-center	Study design	Total patients (n)	Patients Control Group (n)†	Patients Intervention Group (n)§	Patient age (Median/ yr)	Sex (n)	Follow-Up (Months)	Tumor Size (Median/cm)	Immunotherapy drugs regimen
Lin et al^(17)^	2017	China	SC	PS	71	39	32	57.0	M: 36 F: 35	7.4✧(3.6–11.2)	5.01 (Cancer Stage III) and 4.92 (Cancer Stage IV)	Allogeneic Natural Killer Cell
Lin et al^(18)^	2020	China	SC	RCT	62	32	30	62.0	M: 36 F: 26	22	3.9† and 4§	γδ T-cell Infusion
Pan et al^(28)^	2020	China	SC	RCT	92	46	46	57.0	M: 52 F: 40	6–29	4.1† and 4.4§	Natural Killer Cell
He et al^(29)^	2021	China	SC	RS	85	70	15	57.8	M: 38 F: 47	12–70	3.75† and 3.5§	Toripalimab

† Irreversible Electroporation Alone Group;

§ Irreversible Electroporation plus Immunotherapy Group;

✧ Median.

SC: single-center; MC: multi-center; RCT: randomized controlled trial; PS: prospective study; RS: retrospective study; NA: not applicable.

### Endpoints data extraction

The following raw statistics were extracted for data synthesis: hazard ratio (HR) for progression-free survival (PFS), overall survival (OS), carbohydrate antigen 19-9 (CA 19-9), and adverse events (AEs) in both groups. If Kaplan–Meier (KM) curves for PFS and OS were provided instead of HR and 95%CI, time-to-event data were extracted from the Kaplan–Meier curves using WebPlotDigitizer version 4.7 software.^(15)^ Subsequently, this data was used to calculate the HR and 95%CI using the R software package, "IPDfromKM."^(16)^

For CA 19-9, the values from Lin et al.^(17)^ and Lin et al.^(18)^ were extracted using WebPlotDigitizer, version 4.7.^(15)^ Data normality was first assessed using the method reported by Shi et al.^(19)^ Values from Lin et al.^(18)^ were transformed from median and interquartile range to mean and standard deviation using the method described by Wan et al.^(20)^

Progression-free survival was defined as the period from the date of treatment initiation or baseline assessment to objective disease progression, subjective disease deterioration, or death, whichever occurred first. Overall survival was defined as the time from treatment initiation or baseline assessment to death. Progression-free survival was censored on the date of the last cancer assessment if no progression had occurred, and OS was censored at the time of the last follow-up for patient who were alive or lost to follow-up. Serum CA19-9 levels were evaluated at each follow-up using a quantitative sandwich enzyme immunoassay. Adverse events were defined as any unfavorable symptoms or diseases occurring after treatment initiation, including any new health issues or worsening of preexisting conditions, regardless of their relationship to the treatment.

### Quality assessment

We evaluated the risk of bias in the randomized controlled trials (RCTs) using the Cochrane Risk of Bias assessment tool (version 2).^(21)^ Non-randomized studies were assessed using the ROBINS-I ("Risk of Bias in Nonrandomized Studies of Interventions") tool.^(22)^ Two authors independently assessed risk of bias. Disagreements were resolved by consensus after discussing the reasons for discrepancy. Testing for funnel plot asymmetry was not conducted because its power was too low to distinguish between chance and real asymmetry when fewer than 10 studies were included in the meta-analysis.^(23)^

### Statistical analysis

The statistical analysis was conducted using R software, particularly the "meta,""metafor," and "dmetar" packages.^(16)^ For the estimation of meta-analytic measures, an inverse variance estimator was used in a random-effects model. For the estimation of between-study variances (τ^2^) and considering the odds ratio, the Restricted Maximum Likelihood (REML) method^(24)^ was applied. For the HR, the DerSimonian–Laird method^(25)^ was applied. A p<0.05 was considered as the threshold of statistical significance. The results are presented as pooled estimates with 95%CI and plotted as forest plots.^(16,26)^

Heterogeneity was assessed using the I2 statistic^(27)^ and Q-test.^(26)^ Sensitivity analysis was conducted by omitting one study from each analysis to evaluate the effect of each study on the overall result. The extracted data are summarized in tables 2 and 3.

**Table 2:** Clinical outcomes of patients included in each study

Study	HR OS	LLHR OS	ULHR OS	HR PFS	LLHR PFS	ULHR PSF	CA 19-9 Mean Exp	CA 19-9 SD Exp	CA 19-9 Total Exp	CA 19-9 Mean Control	CA 19-9 SD Control	CA 19-9 Total Control
Lin et al^(17)^	0.542	0.335	0.878	0.643	0.394	1.050	77.51	5.47	32	105.78	5.47	39
Lin et al^(18)^	0.550	0.320	0.940	0.580	0.340	0.990	102.10	29.06	30	169.70	65.78	32
Pan et al^(28)^	NA	NA	NA	NA	NA	NA	359.10	41.20	46	475.60	49.40	46
He et al^(29)^	0.301	0.092	0.985	0.274	0.099	0.759	NA	NA	NA	NA	NA	NA

Exp: Experimental Group (IRE plus immunotherapy); Control: Control Group (IRE alone); HR: hazard ratio; OS: overall survival; UL: upper limit; LL: lower limit; PFS: progression-free survival; NA: not applicable; SD: standard deviation.

**Table 3:** Adverse events of patients included in each study

	Overall analysis – random effects model				
Secondary outcomes	n	Estimate (95% CI)	I^2^ (%)	p value*	p value†
Nausea and vomiting	3	OR 1.58 (0.71–3.49)	0	0.26	0.93
Gastroparesis	3	OR 0.88 (0.23–3.40)	0	0.86	0.58
Loss of appetite	2	OR 1.34 (0.48–3.75)	0	0.58	0.80
Diarrhea	2	OR 0.85 (0.25–2.86)	0	0.80	0.65
Pancreatitis	2	OR 1.45 (0.37–5.73)	0	0.60	0.68
Abscess	2	OR 1.12 (0.25–5.06)	0	0.89	0.95
Pain	2	OR 0.66 (0.15–2.85)	0	0.58	0.49
Portal vein thrombosis	2	OR 0.83 (0.11–6.51)	0	0.86	0.80
Cardiac arrythmias	2	OR 0.50 (0.08–3.32)	0	0.47	0.97

* p value for effect,

† p value for heterogeneity.

OR: odds ratio; 95%CI: 95% confidence interval; I^2^: statistical assessment of heterogeneity.

## RESULTS

### Study selection

**Figure 1:**
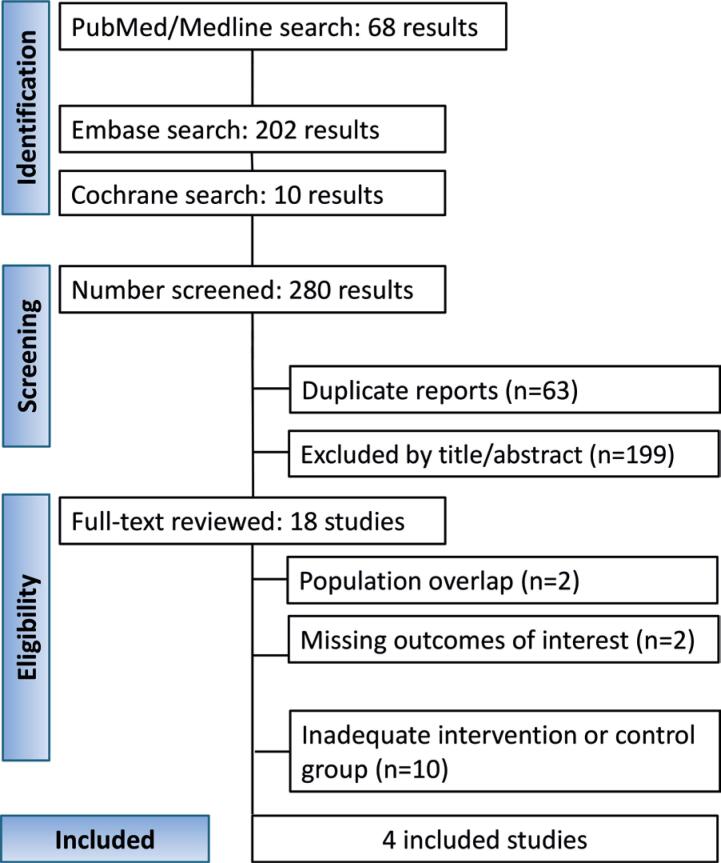
PRISMA flow diagram of study screening and selection

The initial search identified 280 studies. After excluding 63 duplicates, 199 studies were excluded based on their titles and abstracts. Eighteen of the remaining studies were read in full. Among these, 14 were excluded because of population overlap, missing outcomes of interest, or inadequate Intervention or Control Groups This process resulted in four articles^(17,18,28,29)^ deemed eligible for analysis (Figure 1).

### Characteristics of included studies

The selected studies resulted in a sample of 310 patients, with 187 in the Control Group and 123 in the Intervention Group. Among these patients, 48% were female and the mean age was 58.45 years. All the studies were conducted at different hospitals in China. The selected studies were published between 2017 and 2021. Two studies were RCTs, one was an observational retrospective study and the other was a prospective study. All studies were conducted at a single center. The median tumor diameter was 4.0cm in the Control Group and 4.2cm in the Intervention Group. The mean follow-up time was 17.5 months. The immunotherapy drug regimen was the same for the Interventional and Control Groups, but varied across studies. The characteristics of the individual studies are presented in table 1.

### Main findings and heterogeneity of PFS, OS, CA 19-9, and AEs

**Figure 2:**
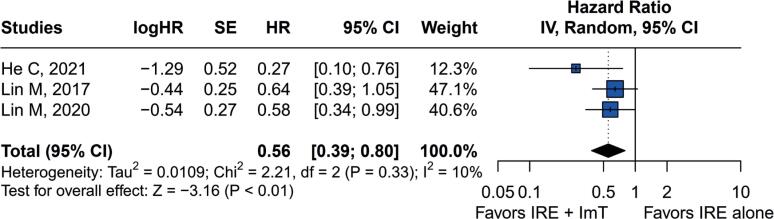
Forest plot for progression-free survival

HR: hazard ratio; SE: standard error; 95%CI: 95% confidence interval; IRE: irreversible electroporation; ImT: immunotherapy.

Pooled PFS indicated that the combination of IRE with immunotherapy effectively protected patients from disease progression compared to IRE alone with an HR of 0.56 (95%CI=0.39–0.80; p<0.01). Heterogeneity was not significant (I^2^=10%, p=0.33 (Figure 2).

**Figure 3:**
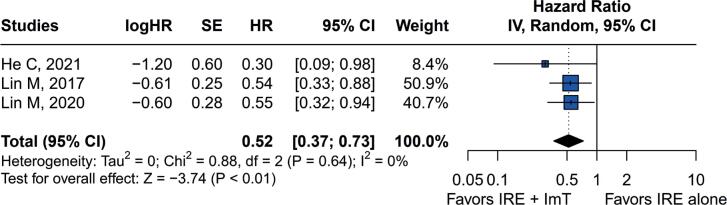
Forest plot for overall survival

HR: hazard ratio; SE: standard error; 95%CI: 95% confidence interval; IRE: irreversible electroporation; ImT: immunotherapy.

Combination therapy of IRE and immunotherapy improved OS with a pooled HR of 0.52 (95%CI=0.37–0.73; p<0.01). No obvious heterogeneity was observed (I^2^=0%, p=0.64 (Figure 3).

**Figure 4:**
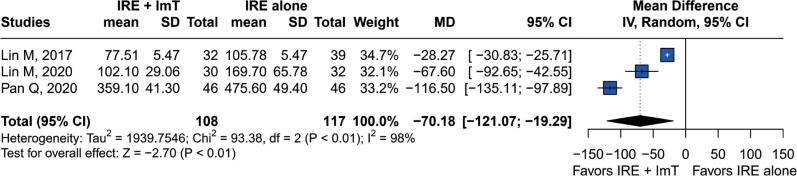
Forest plot of mean differences in CA 19-9 levels

HR: hazard ratio; SE: standard error; 95%CI: 95% confidence interval; IRE: irreversible electroporation; ImT: immunotherapy.

The CA 19-9 outcome was reported in three studies. The pooled results showed significantly lower levels of CA 19-9 in patients receiving IRE and immunotherapy compared to those receiving IRE alone (mean difference [MD]: −70.18 U/L; 95%CI=-121.07 – −19.29; p<0.01). However, the heterogeneity was considerable, with I^2^=98% and p<0.01 (Figure 4). This heterogeneity might be explained by the different CA 19-9 testing intervals implemented in each study during the follow-up period. Lin et al.^(18)^ reported results for CA 19-9 on day 90 after the intervention; Lin et al.^(17)^ reported results on days 1, 7, and 30; and Pan et al.^(28)^ reported CA 19-9 levels on days 1, 7, and 30. Lin et al. and Pan et al. found that CA 19-9 levels remained high on days 1 and 7, but dropped by day 30 in both groups. All three studies reported that CA 19-9 levels were lower in the IRE plus immunotherapy group than in the IRE alone group on days 30 or 90. This heterogeneity might also be attributed to differences in the baseline levels of CA 19-9 in patients before intervention in each study.

**Figure 5:**
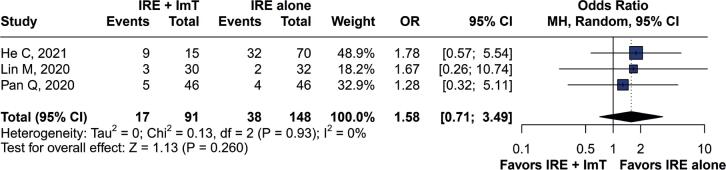
Forest plot of nausea and vomiting as an adverse event

OR: odds ratio; 95%CI: 95% confidence interval; IRE: irreversible electroporation; ImT: immunotherapy; MH: Mantel–Haenszel.

**Figure 6:**
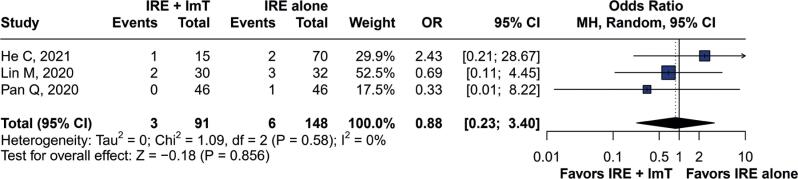
Forest plot of gastroparesis as an adverse event

OR: odds ratio; 95%CI: 95% confidence interval; IRE: irreversible electroporation; ImT: immunotherapy; MH: Mantel–Haenszel.

The overall analysis of AEs is presented in table 3, which shows no significant differences in AEs between groups. No significant difference in the occurrence of AEs such as nausea and vomiting (OR=1.58; 95%CI=0.71–3.49; p=0.26) (Figure 5) and gastroparesis (OR=0.88; 95%CI=0.23–3.40; p=0.85) (Figure 6) was observed between the groups. No heterogeneity was found, as indicated by I^2^=0% and p=0.93 for nausea and vomiting, and I^2^=0% and p=0.58 for gastroparesis. No deaths related to the procedure occurred during follow-up.

### Quality of the studies

Quality ratings for the included studies ranged from "serious risk of bias" to "some concerns." Pan et al.^(28)^ reported "some concerns" regarding the overall risk of bias due to potential issues with the blinding of participants, personnel, and outcome assessors. Lin et al.^(18)^ also reported "some concerns" related to the lack of explicit mention of allocation concealment in the randomization process, the potential for performance bias given the nature of the intervention, and the potential for detection bias from assessors aware of the intervention.^(21)^ Lin et al.^(17)^ and He et al.^(29)^ presented a "serious risk of bias" owing to confounding factors, as at least one confounder was not adequately measured.^(22)^ Individual appraisals of each study included in the meta-analysis are shown in (Table 2S and 3S, Supplementary Material).

## DISCUSSION

This systematic review and meta-analysis evaluated the efficacy and safety of percutaneous IRE combined with immunotherapy compared with IRE alone in patients with advanced LAPC. The findings indicate that combining IRE with immunotherapy significantly improves survival in patients with LAPC compared with IRE alone, and without a significant increase in AEs.

Several factors may contribute to the overall efficacy of the combined therapy, including modulation of the tumor microenvironment, suppression of tumor growth, and enhanced immunomodulatory responses through synergistic therapeutic effects. Breakthroughs in immunotherapy with immune checkpoint inhibitors (ICIs) have dramatically transformed treatment paradigms for other hard-to-treat malignancies, such as melanoma and lung cancer.^(30,31)^ However, the efficacy of immunotherapy remains limited because of the immunosuppressive nature of pancreatic cancer.

Previous studies have demonstrated that IRE promotes M1 macrophage polarization, increases PD-1+ T cells, reduces Tregs cells, and induces *in situ* release of tumor-specific antigens after the procedure further enhancing the efficacy of immunotherapy in patients with pancreatic cancer.^(32,33)^ In addition, one study suggested that the systemic antitumor immune response triggered by IRE can be further enhanced by stimulating the innate immune system with a toll-like receptor-7 (TLR7) agonist and the adaptive immune system with anti-PD-1 checkpoint blockade.^(34)^ A preclinical study by Zhao et al.^(35)^ demonstrated that IRE can reprogram the immunosuppressive tumor microenvironment in PDAC. Their murine PDAC model showed that IRE induced immunogenic cell death, fostered dendritic cell activation, and preserved critical stromal collagen scaffolding. Notably, when combined with anti-PD-1 therapy, IRE facilitated robust CD8^+^ T-cell infiltration, markedly prolonged survival, and even generated long-term immune memory.

The immunotherapy applied in the experimental groups included in this review comprised γδ T-cell infusion, allogeneic natural killer (NK) cell therapy, and Toripalimab.^(17,18,28,29)^ T cells are key components of the tumor microenvironment and previous reports have indicated that γδ T-cells contribute to tumor immune surveillance against various types of tumors.^(36,37)^ NK cells recognize non-self-histocompatibility antigens on cell surface through their NK cell immunoglobulin-like receptors (KIRs).^(38)^

Toripalimab is a monoclonal antibody that targets the PD-1 receptor on T cells and is classified as an ICI. Low PD-1 expression within the pancreatic microenvironment may partially explain the limited response to ICIs; however, this limitation may be offset by the systemic adaptive immune response triggered by IRE, thereby sensitizing tumors to ICI therapy.^(39)^ He et al.^(29)^ demonstrated an increase in CD4+ T helper and CD8+ T cytotoxic cells and a decrease in CD8+ Treg cells in patients treated with IRE and Toripalimab. In addition, elevated levels of cytokines, including IL-4, IL-6, IL-10, TNF, and IFN-γ were observed in the IRE and Toripalimab group.^(29)^ Collectively, these therapies appear to enhance the efficacy of IRE through synergistic effects.

The levels of CA 19-9 were also significantly lower in the group treated with IRE combined with immunotherapy. Studies have shown that lower levels of CA 19-9, or its decrease during systemic treatment, are associated with better outcomes in patients with PDAC.^(40,41)^ Carbohydrate antigen 19-9 is synthesized by normal pancreatic and biliary ductal cells and by gastric, colon, endometrial, and salivary epithelia. It is typically present in small amounts in the serum and levels increase in plasma in neoplastic diseases.^(42)^ The literature suggests that CA 19-9 has an average sensitivity of 81% and a specificity of 90% for pancreatic cancer.^(43)^ However, elevated CA 19-9 levels alone do not always indicate the presence of cancer or advanced disease. This finding may also be due to inflammatory conditions such as pancreatitis and other benign gastrointestinal diseases. Furthermore, CA 19-9 is undetectable in individuals who are Lewis antigen-negative.^(44)^

The results also demonstrated no significant difference in AEs between the groups, indicating that combined therapy does not increase treatment-related risk, aligning with various other studies showing that IRE is a safe treatment for patients with pancreatic cancer.^(45–47)^ The safety of immunotherapy in the treatment of pancreatic cancer has also been evaluated in previous studies.^(48,49)^ Together, these findings supporting the safety and feasibility of combined therapy may facilitate future advances in involving novel regimens and therapeutic agents. Intratumoral immunotherapy, which has a different safety profile, was not included in this review.^(50)^

This meta-analysis had several limitations. The included in the studies all had small sample sizes, with fewer than 100 patients each. Given the limited number of published studies on this topic, observational studies were included alongside RCTs. Additionally, the RCTs did not report whether they were open-label or blind. Variations in IRE techniques and incomplete reporting of IRE parameters, as well as differences in the types of immunotherapy used in each study, may have affected the outcomes. All included studies were conducted in China, meaning that publication bias cannot be rules out, and therefore, the generalizability of the findings to other populations is limited. These results must be interpreted with caution, and large-sample multi-center RCTs are needed to confirm the efficiency of IRE plus immunotherapy in LAPC.

## CONCLUSION

The findings of this systematic review and meta-analysis suggest that combining percutaneous irreversible electroporation with systemic immunotherapy may provide a safe and effective treatment option for locally advanced pancreatic cancer, with irreversible electroporation potentially enhancing the efficacy of systemic immunotherapy in combined applications.

## Data Availability

The underlying content is contained within the manuscript.
