# Supplementary material for: Irreversible electroporation combined with immunotherapy versus irreversible electroporation alone for locally advanced pancreatic cancer: a systematic review and meta-analysis
Source: Einstein (Sao Paulo). 2026 Apr 7;24:eRW1656. doi: 10.31744/einstein_journal/2026RW1656 (PMC13128226; doi:10.31744/einstein_journal/2026RW1656)
Supplement: SUPPLEMENTARY MATERIAL [file 2317-6385-eins-24-eRW1656-suppl01.pdf]

SUPPLEMENTARY MATERIAL

Irreversible electroporation combined with immunotherapy *versus* irreversible electroporation alone for locally advanced pancreatic cancer: a systematic review and meta-analysis

Miriana Mariussi, Laura Costa de Oliveira Lima, Mariano Gallo Ruelas, Victor Arthur Ohannesian, Bruno Murad, Guilherme Strieder de Oliveira, Luiza Giuliani Schmitt, Giovanni Brondani Torri, Stephan Altmayer, Priscila Mina Falsarella, Pedro Luiz Serrano Usón Junior, Rodrigo Gobbo Garcia

DOI: 10.31744/einstein_journal/2026RW1656

Table 1S. Search strategy per database

Database	Search Strategy
Pubmed/Medline (68)	("irreversible electroporation" OR ire OR electroporation) AND (immunotherapy OR "allogenic Vγ9Vδ2 T cells" OR "natural killer cells" OR "Toll-Like Receptor Ligand" OR "IMO-2125" OR nivolumab OR toripalimab OR "Anti-PD-1 Antibody" OR "PD-1/PD-L1 blockade" OR "Immune Checkpoint" OR "Target Therapy" OR pembrolizumab OR keytruda OR dostarlimab-gxly OR larotrectinib OR entrectinib) AND ("locally advanced pancreatic cancer" OR lapc OR "pancreatic cancer" OR "pancreatic neoplasms" OR "advanced pancreatic carcinoma" OR "pancreatic ductal adenocarcinoma" OR pdac)
Embase (202)	("irreversible electroporation" OR ire OR electroporation) AND (immunotherapy OR "allogenic Vγ9Vδ2 T cells" OR "natural killer cells" OR "Toll-Like Receptor Ligand" OR "IMO-2125" OR nivolumab OR toripalimab OR "Anti-PD-1 Antibody" OR "PD-1/PD-L1 blockade" OR "Immune Checkpoint" OR "Target Therapy" OR pembrolizumab OR keytruda OR dostarlimab-gxly OR larotrectinib OR entrectinib) AND ("locally advanced pancreatic cancer" OR lapc OR "pancreatic cancer" OR "pancreatic neoplasms" OR "advanced pancreatic carcinoma" OR "pancreatic ductal adenocarcinoma" OR pdac)
Cochrane Library (10)	("irreversible electroporation" OR ire OR electroporation) AND (immunotherapy OR "allogenic Vγ9Vδ2 T cells" OR "natural killer cells" OR "Toll-Like Receptor Ligand" OR "IMO-2125" OR nivolumab" OR toripalimab OR "Anti-PD-1 Antibody" OR "PD-1/PD-L1 blockade" OR "Immune Checkpoint" OR "Target Therapy" OR pembrolizumab OR keytruda OR dostarlimab-gxly OR larotrectinib OR entrectinib) AND ("locally advanced pancreatic cancer" OR lapc OR "pancreatic cancer" OR "pancreatic neoplasms" OR "advanced pancreatic carcinoma" OR "pancreatic ductal adenocarcinoma" OR pdac)

Table 2S. Risk of bias summary for randomized studies (RoB 2)

Study	Bias from randomization process	Bias due to deviations from intended interventions	Bias due to missing outcome data	Bias in measurement of the outcomes	Bias in selection of the reported result	Overall risk of bias
Pan Q. 2020	some concerns	low	low	some concerns	low	some concerns
Lin M. 2020	some concerns	low	low	some concerns	low	some concerns

Table 3S. Risk of bias summary for non-randomized studies (ROBINS-I)

Study	Bias due to confounding	Bias in selection of participants	Bias in classification of interventions	Bias due to deviations from intended interventions	Bias due to missing data	Bias in measurement of outcomes	Bias in selection of the reported result	Overall risk of bias judgment
He C. 2021	serious	moderate	low	low	low	moderate	low	serious
Lin M. 2017	serious	moderate	moderate	low	low	Low	low	serious
